# Gancao Xiexin decoction combined with mesalazine in the treatment of ulcerative colitis: A protocol for a systematic review and meta-analysis

**DOI:** 10.1097/MD.0000000000023038

**Published:** 2020-11-20

**Authors:** Chun Zhong, Xiaoming Cheng, Bo Jia, PeiYu Xiong, Jinhua Lu, Peixu Zhang, Xinglong Liu, Yunhui Chen

**Affiliations:** a School of Basic Medical Science, Chengdu University of Traditional Chinese Medicine, Chengdu; b XiangYang Hospital of Traditional Chinese Medicine, Xiangyang, China.

**Keywords:** Gancao Xiexin decoction, mesalazine, meta-analysis, protocol, systematic review, ulcerative colitis

## Abstract

**Backgroud:**

Ulcerative colitis (UC) is a chronic inflammatory disease that involves the rectum, colon and ileum. Gancao Xiexin decoction (GCXXD) is a classic herbal formula in *Shanghanlun*. More and more research evidence shows that GCXXD has a certain therapeutic effect on UC. Therefore, we designed this study protocol aim to evaluate the efficacy and safety of GCXXD combine with mesalazine for UC.

**Methods:**

We will systematically search 6 databases, including PubMed, the Cochrane Library, EMBASE, CNKI, VIP, Wang-fang database up to July 2020 to obtain eligible studies. The primary outcomes will focus on the clinical effectiveness. Review Manager 5.3 software will be used for data analysis.

**Results:**

This study will provide the systematic evidence of UC treated with GCXXD combine with mesalazine.

**Conclusion:**

The findings of this meta-analysis will provide evidence to judge whether GCXXD combine with mesalazine is a more effective intervention compare to mesalazine only for patient of UC.

**INPLASY registration number:**

INPLASY202080008.

## 1 Introduction

Ulcerative colitis (UC) is a chronic, idiopathic inflammatory disease which is a type of inflammatory bowel disease (IBD), most commonly developing patients aged 30 to 40 years old and giving rise to disability.^[1,2]^ The main characteristics of UC include continuous and diffuse inflammation that is involving the rectum, colon, and ileum.^[3]^ Epidemiological studies have shown that prevalence of 286 cases per 100,000 population in the United States, 505 of 100,000 in Norway, and a low of 6.67 per 100,000 in Malaysia,^[4]^ meanwhile, the incidence of UC have been increasing in Asia. So far, the etiology and pathophysiology of UC remain unclear, the symptoms of UC not only include gastrointestinal symptoms, such as bloody diarrhea, abdominal pain, fecal urgency, and/or tenesmus,^[5]^ but also include extraintestinal manifestations such as joint, eyes, skin mucosa, hepatobiliary, and bone.^[6]^ The objectives for UC are clinical and endoscopic remission for induction and maintenance. Patients of UC may be up to more than 10 bowel movements during onsets of the disease, thus strongly affecting the quality of life,^[7]^ this is being a huge burden of health and economy for patients themselves and countries as well, even become an important public health problem worldwide.^[8]^

There are several therapeutic armamentaria used to treat UC: aminosalicylates, corticosteroids, immunomodulators, and surgery to resect the colon.^[9,10]^ Mesalazine among them is often applied in the initial treatment of UC,^[11]^ but it can lead to serious adverse effects like other drugs. Therefore, it is required to seek more effective treatment.

Recently, traditional Chinese medicine (TCM) plays an important role in preventing and treating some diseases including UC, and gradually has been received worldwide. Gancao Xiexin Decoction (GCXXD) was from Shanghanlun wrote by Zhang Zhongjing (150-219CE), could be used for the treatment of UC, whose herbs consists of Gancao (Glycyrrhiza Uralensis), Huangqin (Scutellaria Baicalensis), Ganjiang (Rhizoma Zingiberis), Banxia (Pinellia), Dazao (Ziziphus Jujube), Huanglian (Coptis). Animal experiments have shown that GCXXD could improve the pathological changes of the colon, decrease the level of IL-6 in colon tissue, inhibit the activation of STAT3 in rats with UC,^[12]^ and TLR4/NF-κB signaling pathway.^[13]^ Clinical pieces of research have also shown that GCXXD combined with mesalazine has achieved a good curative effect in the treatment of UC.^[14,15]^ However, there is no systematic review and meta-analysis have been regarding GCXXD combine with mesalazine for UC. Therefore, we are writing this protocol for a systematic review and meta-analysis to value its effectiveness and safety, which provide systematic evidence.

## 2 Methods

### 2.1 Protocol registration

This protocol had been registered with the International Platform of Registered Systematic Review and Meta-Analysis Protocols (INPLASY), the registration number is INPLASY202080008, and the doi number is 10.37766/inplasy2020.8.0008. This study will be followed the guidelines of Preferred Reporting Items for Systematic Review and Meta-Analysis Protocols (PRISMA-P).^[16]^

### 2.2 Inclusion criteria

#### 2.2.1 Types of study

Randomized controlled trials (RCTs) will be included for systematic review and meta-analysis without limitations on publication type.

#### 2.2.2 Participants

We will include patients who were clinically diagnosed with UC regardless of sex, age, ethnicity, economic status, or education. We will use the diagnostic criteria of UC from the Chinese consensus on diagnosis and treatment of IBD (Beijing, 2018).^[17]^

#### 2.2.3 Types of intervention

The experimental group was treated with GCXXD combined with mesalazine; the control group was treated with mesalazine alone.

### 2.3 Type of outcomes

#### 2.3.1 Primary outcomes

The primary outcomes of this meta-analysis will focus on clinical effectiveness. The clinical efficacy rate was divided into the following categories according to the degree of improvement of the patient: (1) remission: clinical symptoms disappeared, colonoscopy showed that the mucosa was generally normal or no active inflammation. (2) Effective: the clinical symptoms disappeared, and colonoscopy showed mild inflammation of the mucosa. (3) Ineffective: there was no improvement in clinical symptoms and colonoscopy.

#### 2.3.2 Secondary outcomes

The secondary outcome includes the level of inflammatory cytokines such as IL-6, IL-10, IL-17, TNF-α, and so on, and the modified Mayo scores^[18]^ of UC.

### 2.4 Search strategy

In this meta-analysis, we searched PubMed, Cochrane Library, EMBASE, CNKI, VIP, Wang-fang database according to the recommendations of the Preferred Reporting Items for Systematic Reviews and Meta-analyses Statement.^[19]^ The computer will include RCTs on GCXXD combine with mesalazine for UC published from the establishment to July 31, 2020, and retrieved in English or Chinese. The following key terms are used: GCXXD, mesalazine, UC, and RCTs. The retrieval strategy varies according to the different databases. The process of search is shown in Table 1.

**Table 1:** The search strategy in PubMed.

Ulcerative colitis[Mesh]
Ulcerative colitis[Title/Abstract]
Inflammatory bowel disease
1 or 2 or 3
Mesalamine[Mesh]
Mesalazine[Title/Abstract]
m-Aminosalicylic Acid[Title/Abstract]
5-Aminosalicylic Acid[Title/Abstract]
meta-Aminosalicylic Acid[Title/Abstract]
Asacol[Title/Abstract]
Asacol[Title/Abstract]
Ascolitin[Title/Abstract]
Ascolitin[Title/Abstract]
Ascolitin[Title/Abstract]
Fivasa[Title/Abstract]
Salofalk[Title/Abstract]
Lixacol[Title/Abstract]
Mesalamine Hydrochloride[Title/Abstract]
Hydrochloride, Mesalamine[Title/Abstract]
Mesalamine Monosodium Salt[Title/Abstract]
Monosodium Salt, Mesalamine[Title/Abstract]
Mesasal[Title/Abstract]
Novo-5 ASA[Title/Abstract]
Pentasa[Title/Abstract]
Rowasa[Title/Abstract]
5-Aminosalicylate[Title/Abstract]
5 or 6 or 7 or 8 or 9 or 10 or 11 or 12 or 13 or 14 or 15 or 16 or 17 or 18 or 19 or 20 or 21 or 22 or 23 or 24 or 25 or 26
Gancao Xiexin Decoction[Mesh]
Gancao Xiexin Decoction[Title/Abstract]
28 or 29
Randomized Controlled Trial[Publication Type]
Randomized[Title/Abstract]
31 or 32
4 and 27 and 30 and 33

### 2.5 Studies selection

**Figure 1:**
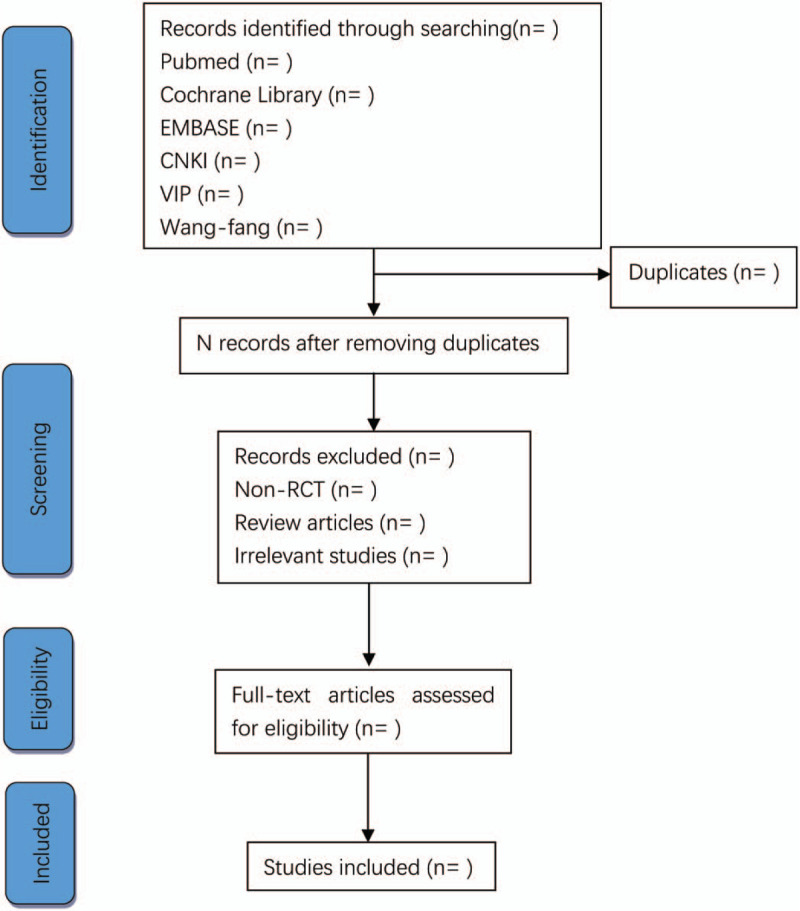
Flow chart of the study selection.

The study selection will be conducted by 2 researchers independently according to the inclusion criteria. All results should be imported into Endnote X9, and duplicates will be removed. Researchers will read the headings and abstracts to exclude irrelevant pieces of literature (reasons for any excluded studies will be written), and then read the full text that consistent with the inclusion criteria to determine whether including this study. If there was a difference between the 2 authors during the selection process, we will be solved by a discussion with a third author together. The process of this meta-analysis will have emerged as the PRISMA flow diagram (Fig. 1).

### 2.6 Data extraction and management

Two investigators extracted the data separately by a standardized extraction form. The data to be extracted will include title, author, year, gender, age, sample size, interventions, outcomes, etc. Any disagreement will be resolved through discussion within the group.

### 2.7 Assessment of risk of bias

The qualities in the included studies were assessed by 2 investigators using the Cochrane risk of bias tool which consists of the following items of bias related to the quality of RCTs: random sequence generation, allocation concealment, blinding of participants, blinding of personnel, blinding of outcome assessment, incomplete outcome data, selective outcome reporting, and other potential biases.

### 2.8 Data synthesis

Data synthesis will be finished using RevMan 5.3 software when the meta-analysis was allowed for the included studies. We will estimate the heterogeneity of enrolled literature by *I*^2^ tests. If the data had high heterogeneity (*P* < .1 or *I*^2^ > 50%), we chose a random-effects model for analysis, or a fixed-effects model was selected. Odds ratio will be used to assess the efficacy of GCXXD combine with mesalazine compare to mesalazine monotherapy. Mean difference will be calculated for continuous outcomes, and a 95% confidence interval was given. If there is high heterogeneity, we will carry out a subgroup analysis and sensitivity analysis to explore potential causes. A funnel plot will be used to examine publication bias when more than 10 trials in the meta-analysis.

### 2.9 Grading the quality of evidence

The Grading of Recommendations Assessment, Development and Evaluation (GRADE) will be used for evaluating the quality level of evidence.

### 2.10 Ethics

Ethical approval was not necessary, for this article is not involve personal information.

## 3 Discussion

UC has become a common and frequently-occurring disease nowadays. Not only the global burden of UC but also the healthcare and societal costs continue to rise. The mainstream therapy is drug therapy, but long-term use will have a potential risk of side effects. Besides, the prognosis of the disease is not optimistic. Although there is no appearance of UC in ancient TCM literature, according to the similar symptoms and characteristics of the disease, which attributes to the category of dysentery, etc. GCXXD was used to treat the syndrome of epigastric lumpy stiffness, modern pharmacological studies have found that GCXXD could regulate gastric mucosal secretion, anti-ulcer, and other effects.^[20]^ It is widely used in the immune system regulating immune function^[21]^ and digestive system diseases.^[22,23]^ Clinical studies show that GCXXD can relieve UC, and achieve obvious curative effects.^[24]^ However, the effectiveness and safety of GCXXD in the treatment of UC are still uncertain. Therefore, it is necessary for a systematic review to provide objective evidence of GCXXD in treating UC. Of course, there is still some potential weakness in this meta-analysis, we hope to provide a powerful reference for researchers and clinicians.

## Author contributions

**Conceptualization:** Xinglong Liu, Yunhui Chen, Bo Jia.

**Data curation:** Chun Zhong.

**Investigation:** Jinhua Lu, Xinglong Liu.

**Methodology:** Peiyu Xiong, Peixu Zhang.

**Project administration:** Chun Zhong, Xiaoming Cheng.

**Software:** Xiaoming Cheng.

**Supervision:** Xinglong Liu, Yunhui Chen, Bo Jia.

**Validation:** Jinhua Lu, Peixu Zhang.

**Writing – original draft:** Chun Zhong.

**Writing – review & editing:** Xiaoming Cheng, Xinglong Liu, Bo Jia.
